# Prevalence of lower extremity edema following inguinal lymphadenectomy: A systematic review and meta-analysis

**DOI:** 10.1016/j.jpra.2024.11.001

**Published:** 2024-11-17

**Authors:** Brett A. Hahn, Milan C. Richir, Arjen J. Witkamp, Tim de Jong, David D. Krijgh

**Affiliations:** a Department of Plastic and Reconstructive Surgery**,** University Medical Center Utrecht**,** Utrecht**,** The Netherlands; b Department of Oncologic Surgery**,** University Medical Center Utrecht**,** Utrecht**,** The Netherlands; c Department of Plastic and Reconstructive Surgery**,** Radboud University Medical Center**,** Nijmegen**,** The Netherlands

**Keywords:** Lower extremity lymphedema, Inguinal, Lymph node dissection, Lymphadenectomy, Edema, Prevalence

## Abstract

**Background:**

Lower extremity lymphedema (LEL) can develop because of inguinal lymph node dissection in the treatment of gynecologic, genitourinary, and dermatological malignancies. To optimize patient counseling and patient selection for microsurgical interventions aimed at preventing or treating LEL, its prevalence and associated patient characteristics must be accurately documented. This systematic review and meta-analysis provides a comprehensive overview of literature on the reported prevalence of LEL in patients undergoing inguinal lymphadenectomy.

**Methods:**

From Embase, PubMed, and Web of Science databases, 23 studies were identified that met the inclusion criteria. This review was conducted in accordance with the preferred reporting items for systematic reviews and meta-analyses guidelines. Risk of bias was assessed using the Risk of Bias in Non-randomized Studies-of Exposure tool.

**Results:**

Random-effects meta-analyses of proportions estimated a 24% (95% confidence interval [CI]: 17-31) pooled prevalence of LEL with a high degree of heterogeneity between the studies (*I^2^*=96%, *p* < 0.01). Subgroup analysis revealed significant differences in LEL prevalence based on the indications for inguinal lymphadenectomy. The pooled LEL prevalence was 25.75% (95% CI: 0.00-96.16) for patients who underwent lymphadenectomy for melanoma, 12.22% (95% CI: 1.03-23.40) for penile cancer, 30.96% (95% CI: 21.08-40.84) for vulvar cancer, and 13.62% (95% CI: 0.00-51.02) for miscellaneous indications.

**Conclusion:**

The findings from this study emphasize the importance of considering malignancy etiology when assessing the risk of LEL following inguinal lymphadenectomy. This knowledge could aid physicians in informing patients about the risk of LEL, while also facilitating proper patient selection for microsurgical interventions.

## Introduction

Lower extremity lymphedema (LEL) is a common condition that develops following inguinal lymph node dissection, as part of the treatment for gynecologic, genitourinary, and dermatological malignancies.^1^ The accumulation of interstitial fluid from impaired lymphatic drainage often leads to functional disability, pain, and discomfort.^2^^,^^3^ Limitations in the normal range of motion, psychological distress, and social isolation in patients with LEL can significantly impact their quality of life.^4^^,^^5^

Although diagnosis of secondary lymphedema in the lower extremity is typically made through clinical presentation, several measurement techniques and staging definitions have led to a wide range of reported prevalence rates.^6^ Furthermore, methods for measuring the degree of lymphedema, such as circumference measurements of the lower extremity or water displacement, are heterogeneous in consistency and only accurate when properly performed.^7^ Improved patient counseling and thorough follow-up assessment following lymphadenectomy depend on the physicians’ understanding of which patients are at the greatest risk for LEL. Without clear insight into the prevalence of LEL and associated patient characteristics, a physicians’ ability to tailor care and guide decision-making for diverse populations remains limited.

Although the initial management of LEL consists of a combination of physical and compression therapies, the high costs and time-consuming trajectory associated with this approach may weaken patient compliance.^8^, ^9^, ^10^ Furthermore, conservative therapy does not address the underlying cause of LEL. Emerging microsurgical interventions using lymphaticovenous anastomosis (LVA) or vascularized lymph node transfer (VLNT) show promising results in minimizing the degree of edema development.^11^^,^^12^ LVA facilitates the bypass of obstructed lymphatic vessels through anastomoses to nearby venules, whereas VLNT has the potential to restore physiologic lymphatic fluid drainage function of the affected lower extremity.

However, the efficacy of reconstructive procedures at optimizing the physical benefits and reducing the risk of potential complications depends on early detection and diagnosis of lymphedema.^13^^,^^14^ Capturing the true prevalence of LEL is crucial to understand its impact on health care and which patient groups face the greatest risk of developing this condition following inguinal lymphadenectomy. Proper selection of patients who could benefit from microsurgical interventions could also be improved if the physicians are better guided by the true prevalence of LEL and accurately documented associated characteristics. Through a systematic review and meta-analysis, this study aimed to provide a comprehensive overview of published literature on the prevalence of LEL in patients who undergo inguinal lymphadenectomy.

## Methods

### Literature search

A systematic literature review of electronically available publications was performed on April 19, 2023. This review was conducted in accordance with the preferred reporting items for systematic reviews and meta-analyses (PRISMA) guidelines^15^, and was registered in the international prospective register of systematic reviews (PROSPERO) under the registration number: CRD42023462628.^16^ No amendments were made to the registered protocol during the course of this study. Two reviewers (B.H. and D.K.) searched three online databases (Embase, PubMed, and Web of Science) using a search string developed with the help of a librarian (Supplemental File 1). All studies returned from the search were independently reviewed and included if the prevalence of lower extremity edema in patients who underwent an inguinal lymphadenectomy was reported. Studies investigating secondary outcomes of lymphadenectomies other than inguinal were excluded, as were those that reported lymphedema in the upper extremity. Duplicates, conference abstracts, systematic reviews, meta-analyses, case reports, nonclinical studies, non-English studies, and studies with sample sizes less than 40 operated groins were further excluded. A re-run of the original search was conducted prior to submission for publication to ensure that all studies meeting the aforementioned inclusion criteria were included for analysis.

### Data extraction

The following variables were extracted from the included articles into a standardized spreadsheet: title, authors, year of publication, country of origin, study design, sample size, age, body mass index (BMI), sex distribution, lymphadenectomy indication, malignancy stage, (neo)adjuvant therapy, surgical intervention and technique, laterality of lymph node dissection, number of lymph nodes resected, lower extremity lymphedema prevalence, lymphedema definition, data collection and edema measurement methods, follow-up period, and surgical complications. Two reviewers (B.H. and D.K.) independently extracted the data from articles, figures, and tables. Accuracy of entered data, as well as any uncertainties or disagreements were resolved by a third reviewer.

### Quality assessment

The Risk Of Bias In Non-randomized Studies-of Exposure (ROBINS-E) tool was used to assess the quality of the included studies.^17^ Each of the seven domains of bias included in the ROBINS-E tool were addressed using a series of signaling questions that aimed to gather important information on the quality of the study. The risk of bias, predicted direction of bias, and whether the risk of bias sufficiently threatened conclusions about whether the exposure to inguinal lymphadenectomy had a considerable effect on lower extremity edema as an outcome were considered before an overall judgment was made for each article. Studies were classified as having either low, some concerns, high, or very high risk of bias. Risk of bias was visualized using the Risk-of-bias VISualization (robvis) tool.^18^

### Statistical analysis

Random-effects meta-analyses of proportions were performed on all included studies to quantify the prevalence of lower extremity edema following inguinal lymphadenectomy. Forest plots were generated to visualize the heterogeneity between studies. Subgroup analyses were performed using random-effects models to investigate the potential sources of effect size variation between the studies. Studies were pooled into subgroups according to the lymphadenectomy indication, age of participants, saphenous vein-sparing surgical technique, lymph node yield, adjuvant radiotherapy exposure, follow-up period, data collection method, unit of LEL reporting, and study risk of bias. A *Q*-test was used to determine if LEL prevalence differed significantly between the various subgroups. All statistical analyses were performed using the meta package^19^ in R Statistical Software (v4.3.1; R Core Team 2023).^20^ A two-sided *p*-value < 0.05 was considered significant. The dataset and script used to perform analyses are available at: https://osf.io/nd742/?view_only=ac06c0451d9944fd85fb7fe707dd590c.

## Results

### Study characteristics

**Figure 1:**
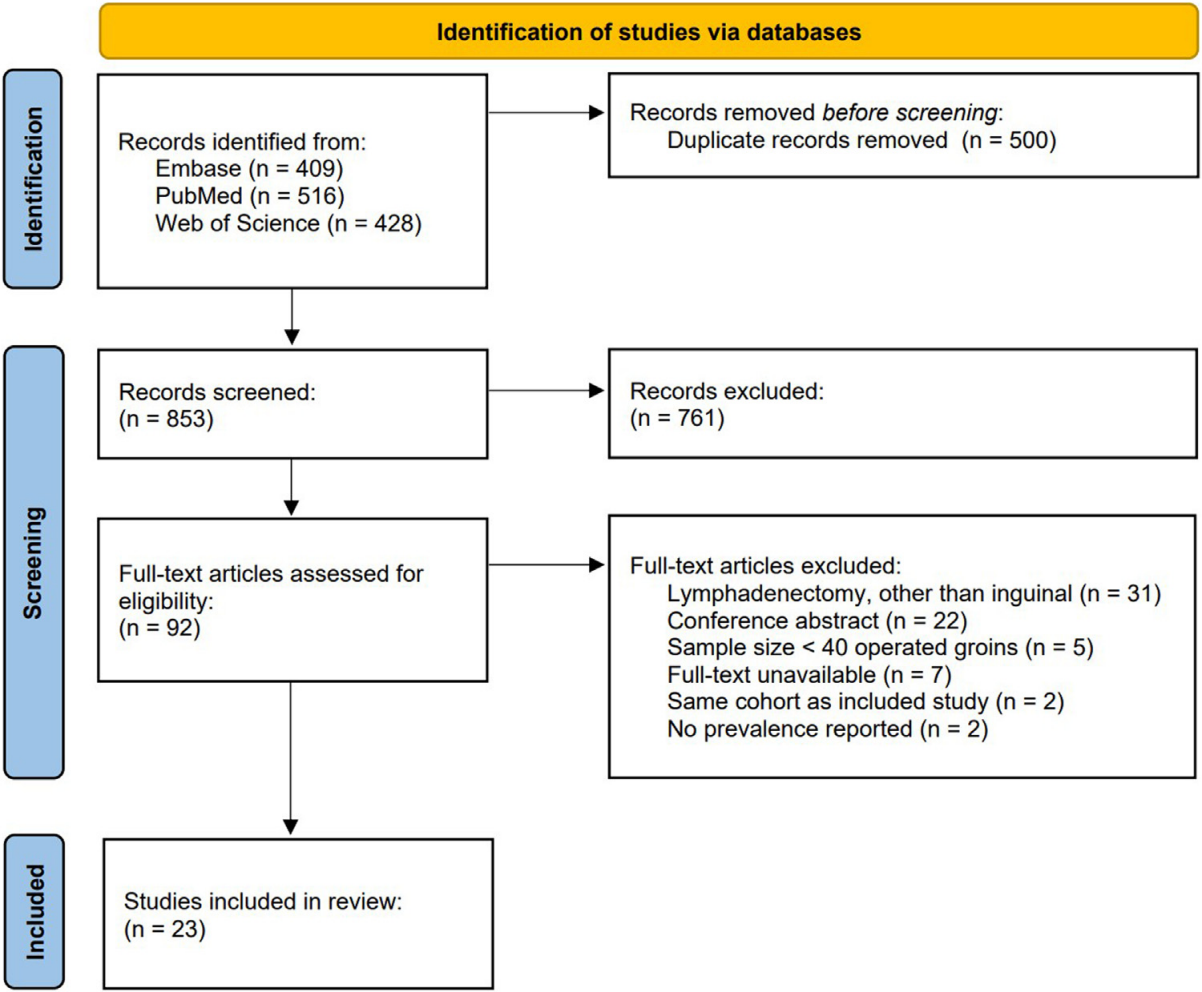
Preferred reporting items for systematic reviews and meta-analyses (PRISMA) flow diagram of systematic database search.

After the removal of duplicates, a total of 853 original articles were identified in PubMed, Embase, and Web of Science databases. Following the initial title/abstract screening, 92 articles underwent full-text review, and 23 articles that met all inclusion and exclusion criteria were included.^21^, ^22^, ^23^, ^24^, ^25^, ^26^, ^27^, ^28^, ^29^, ^30^, ^31^, ^32^, ^33^, ^34^, ^35^, ^36^, ^37^, ^38^, ^39^, ^40^, ^41^, ^42^, ^43^ The search string and review methodology are described in the PRISMA flow diagram in Figure 1.

**Figure 2:**
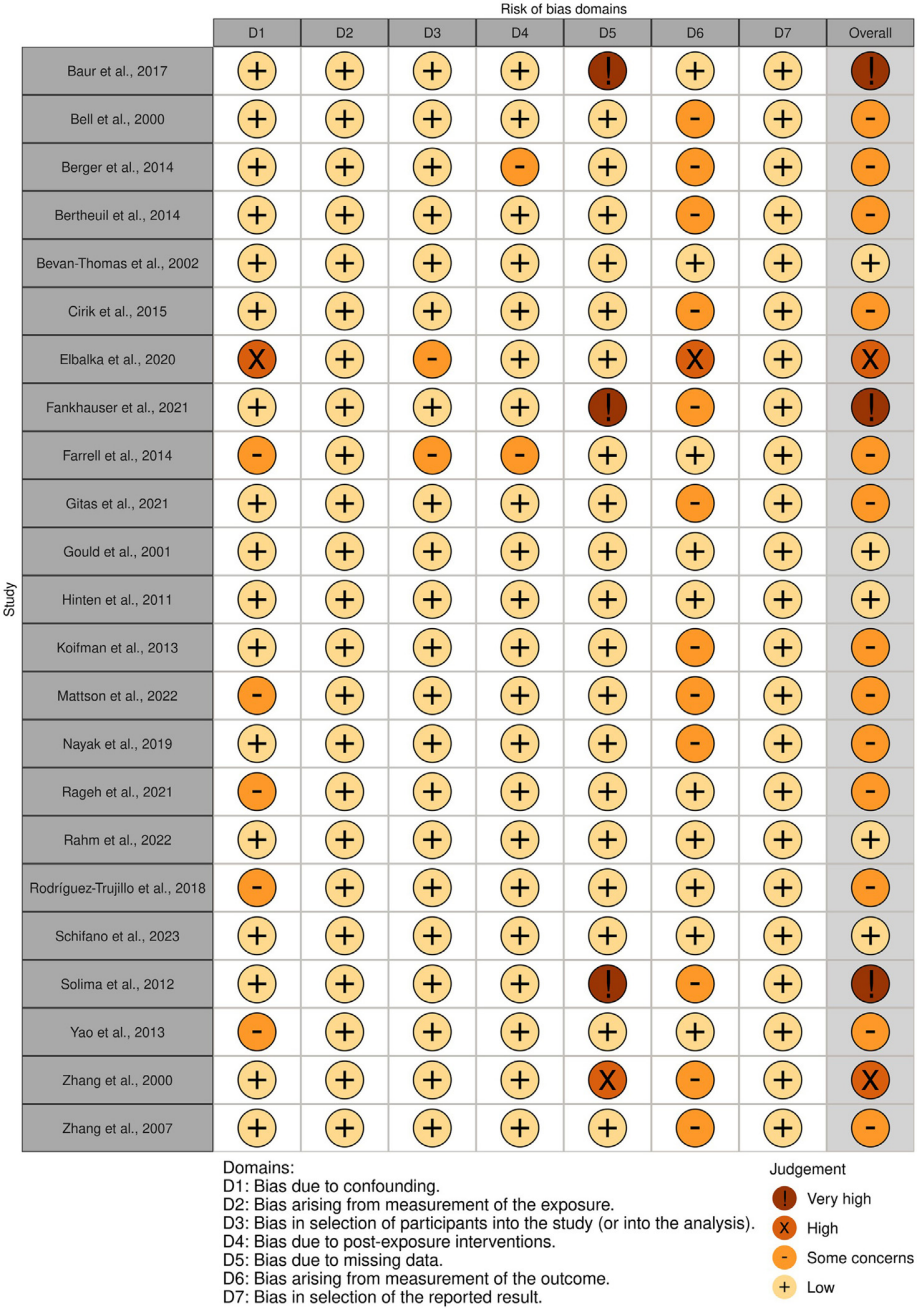
Risk of bias assessment. Overall judgment made on the basis of seven domains (D1-7) in the Risk of Bias in Non-randomized Studies-of Exposure (ROBINS-E) tool. Visualization generated using the Risk-of-bias VISualization (robvis) tool.

All included studies were published between 2000 and 2023, and a total number of 2,376 patients with ages ranging from 23 to 94 years were included in this review (Table 1). Three prospective, 1 cross-sectional, and 19 retrospective studies were included across 13 different countries. Patients underwent inguinal lymphadenectomy for vulvar cancer in 13 studies, penile cancer in 5 studies, melanoma in 2 studies, and for multiple indications in 3 studies. The number of lymph nodes resected during lymphadenectomy varied greatly between studies, while 7 studies did not report nodal yield at all. Five studies carried out inguinal lymphadenectomy using the saphenous vein sparing surgical technique. Patients underwent adjuvant radiotherapy in 10 studies. Most studies reported outcomes with a follow-up period >12 months (n=19). Quality assessment of each study using the ROBINS-E tool identified 3, 2, 13, and 5 studies as very high risk, high risk, some concerns, and low risk of bias, respectively (Figure 2).

**Table 1:** Overview of included studies.

### Prevalence of lower extremity lymphedema

**Figure 3:**
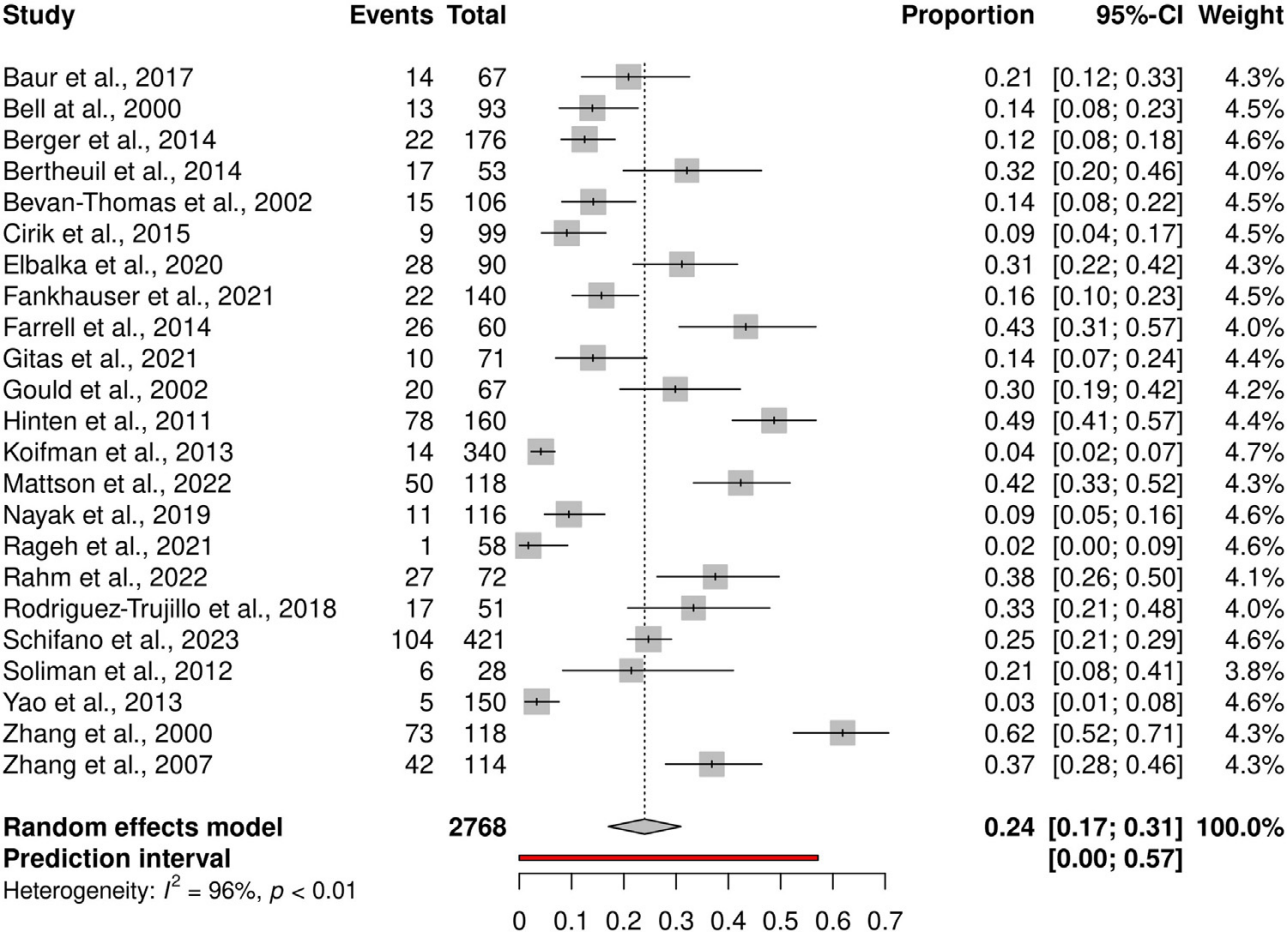
Forest plot of lower extremity lymphedema prevalence.

All studies reported LEL prevalence in the total number of patients who underwent inguinal lymphadenectomy, or the total number of operated groins. The definition of and methods by which LEL was measured or determined differed greatly between the studies. The LEL prevalence reported ranged from 3% to 62% (Figure 3). The pooled prevalence in this meta-analysis was 24% (95% CI: 17-31) with a high degree of heterogeneity among the studies *(I^2^*=96%, *p* < 0.01).

Subgroup analysis revealed significant differences in LEL prevalence based on the indication for inguinal lymphadenectomy (Table 2). The pooled LEL prevalence was 25.75% (95% confidence interval [CI]: 0.00-96.16) for studies in which patients underwent lymphadenectomy for melanoma, 12.22% (95% CI: 1.03-23.40) for penile cancer, 30.96% (95% CI: 21.08-40.84) for vulvar cancer, and 13.62% (95% CI: 0.00-51.02) for miscellaneous indications. No statistically significant differences were found between the subgroups pooled by age, surgical technique, lymph node yield, adjuvant radiotherapy, follow-up period, data collection method, unit of LEL reporting, or study risk of bias. Although the *p*-values of the *Q*-test between age (*p*=0.0506), surgical technique (*p*=0.0551), LEL units (*p*=0.0568), and risk of bias subgroups (*p*=0.0512) were larger than the conventional significance threshold (*p* < 0.05), results still indicate a difference on a trend level.

**Table 2:** Subgroup analyses. Studies pooled according to the indications for lymphadenectomy, age, surgical technique, nodal yield, exposure to adjuvant radiotherapy, follow-up period, data collection method, unit in which LEL was reported, and study risk of bias.

Variable	Subgroup	N studies	LEL prevalence	95% CI	*I^2^*	*p_subgroup_*
Indication	*Melanoma*	2	25.75%	0.00-96.16	47.40%	0.0115^a^
	*Penile cancer*	5	12.22%	1.03-23.40	95.80%	
	*Vulvar cancer*	13	30.96%	21.08-40.84	94.40%	
	*Miscellaneous*	3	13.62%	0.00-51.02	94.20%	
Age	*<65 years*	16	20.56%	13.20-27.92	94.80%	0.0506
	*>65 years*	6	34.53%	14.09-54.97	96.40%	
	*Not reported*	1	13.98%	6.93-21.03	—	
Surgical technique	*Saphenous-sparing*	5	14.19%	2.30-2.60	85.00%	0.0551
	*Saphenous-resecting*	22	24.32%	17.90-30.75	94.80%	
Nodal yield	*<10 nodes*	7	21.78%	9.65-33.90	92.90%	0.7026
	*10 nodes*	2	25.32%	0.00-100.0	91.80%	
	*>10 nodes*	7	19.69%	4.64-34.75	95.90%	
	*Not reported*	7	30.54%	12.32-48.76	97.20%	
Adjuvant radiotherapy	*Yes*	10	27.27%	15.37-39.17	96.30%	0.3957
	*No*	13	21.46%	11.90-31.01	95.90%	
Follow-up	*<12 months*	3	20.05%	0.00-67.22	95.50%	0.4964
	*12 months*	1	32.08%	19.51-44.64	—	
	*>12 months*	19	24.26%	16.36-32.15	96.30%	
Data collection	*Clinical*	2	19.03%	0.00-100.0	98.10%	0.9491
	*Medical records*	19	24.71%	17.32-32.09	94.90%	
	*Questionnaire*	2	23.22%	0.00-100.0	97.30%	
LEL units	*Groins*	13	18.95%	8.96-28.93	96.30%	0.0568
	*Patients*	10	30.81%	21.28-40.33	91.30%	
Risk of bias	*Very high*	3	17.59%	9.83-25.35	0.00%	0.0512
	*High*	2	46.55%	0.00-100.0	95.40%	
	*Some concerns*	13	18.94%	9.66-28.22	94.10%	
	*Low*	5	30.72%	14.32-47.13	91.90%	

^a^*p* < 0.05.

LEL, lower extremity lymphedema.

## Discussion

This systematic review aimed to provide a comprehensive overview of the published literature on LEL prevalence in patients who underwent inguinal lymphadenectomy. Meta-analyses were designed to further elucidate the factors associated with LEL to better characterize patients at greatest risk for developing the condition following surgery. The pooled prevalence of LEL was estimated to be 24% (95% CI: 17-31). Patients who underwent inguinal lymphadenectomy in the treatment for vulvar cancer had higher LEL prevalence (30.96%) than those treated for melanoma (25.75%) or penile cancer (12.22%). Although there was a high degree of heterogeneity among the studies, no statistically significant differences were found in LEL prevalence between subgroups pooled by age, lymph node yield, adjuvant radiotherapy exposure, follow-up period, data collection method, unit of LEL reporting, or study risk of bias.

### Risk factors for lymphedema

The current subgroup analysis showed statistically significant differences in LEL prevalence between patients with various indications for lymphadenectomy based on malignancy etiology. Previous literature has reported similar lymphedema prevalence rates that varied by the type of malignancy independent of treatment.^44^^,^^45^ The risk of developing LEL and potential impact on the quality of life may be important to discuss with patients undergoing lymphadenectomy, especially those with gynecologic malignancies, such as vulvar cancer. Among the 60 patients who underwent lymphadenectomy for vulvar cancer in Farrell et al.,^28^ prevalence of LEL was 43.33%, which was considered present if the patients reported “moderate” or “severe” swelling by means of a questionnaire. Contrarily, Koifman et al.^33^ reported LEL prevalence in 4.12% of patients who underwent lymphadenectomy for penile cancer using questionnaires at follow-up. Data collected on lymphedema from medical records, as done by Hinten et al.^32^ and Mattson et al.,^34^ reported LEL prevalence in 48.75% and 42.37% of patients who underwent lymphadenectomy for vulvar cancer, respectively.

Though not statistically significant, clinical differences were observed between LEL prevalence according to the patients’ age. A greater proportion of patients older than 65 years with lymphedema suggests that they may benefit from microsurgical interventions to minimize the potential impact of secondary lymphedema. Among patients with a median age of 74 years, LEL prevalence following lymphadenectomy was reported by Rahm et al.^37^ at 37.50%. Similarly, Rodríguez-Trujillo et al.^38^ reported LEL prevalence of 33.33% in their patient population with a median age of 71.3 years. However, Hinten et al.^32^ reported younger age as an independent risk factor for developing LEL. This could be explained by the greater change in postoperative movement restrictions experienced by younger women compared to their older counterparts who already had a degree of immobility attributed to other conditions, such as cardiovascular disease.

Surgical variations of inguinal lymphadenectomy, such as saphenous vein-sparing, have been suggested to reduce the postoperational morbidity associated with LEL. Although resection of the saphenous vein has been documented to disrupt collateral lymphatic and vascular channels, the technique remains in practice to achieve optimal oncologic survival outcomes.^48^ Although subgroup analysis revealed no statistically significant difference in LEL between the saphenous-sparing and saphenous-resecting surgical techniques, the lower pooled prevalence among patients with preserved saphenous vein compared to those with resected saphenous vein (14.19% vs. 24.32%) could be clinically relevant. Although the follow-up times were comparable between the five studies that employed the saphenous-sparing technique, the heterogeneity between lymphadenectomy indication calls into question the generalizability of these findings. Several studies^29^^,^^42^^,^^43^ reported statistically significant lower prevalence of LEL in patients who underwent lymph node dissection with the saphenous-sparing technique for penile and vulvar cancer. However, Baur et al.^21^ could not confirm significant differences in LEL attributed to the sparing of the saphenous vein for patients treated for melanoma. The prospective study conducted by Rageh et al.^36^ only implemented the saphenous-sparing technique, and therefore did not have a group to compare LEL prevalence in patients with resected saphenous vein. Though not a primary outcome of this review, patient survival rates were not found in any of the five studies to be closely related to whether the saphenous vein was spared. Furthermore, none of the five studies reported significant differences in local or distant tumor recurrence rates among the patients according to the surgical technique they were subjected to. More direct comparative data on patients undergoing lymphadenectomy for a diverse range of indications are needed to investigate the long-term outcomes of saphenous-sparing surgery on LEL prevalence in relation to oncologic outcomes.

Previous studies identified patients who received radiotherapy following lymphadenectomy as being at a greater risk of developing LEL than those who do not.^46^ Although subgroup analysis demonstrated no statistically significant difference in LEL prevalence among the studies in which the patients were exposed to adjuvant radiotherapy, the pooled prevalence was higher. At an individual study level, Cirik et al.^26^ reported higher but not statistically significant higher LEL prevalence among patients who received adjuvant radiotherapy. Early postoperative infections following lymphadenectomy have also been shown to increase the risk of lymphedema.^47^ Subgroup analysis was not possible as all studies did not report the postoperative complication rates separately for those who did or did not develop LEL. Among all patients who developed LEL, Gitas et al. reported that ≥50% experienced early postoperative complications, such as lymphocele or wound dehiscence. Whereas Soliman et al.^40^ found that patients with early LEL often had wound cellulitis in the same groin, such postoperative complications did not correlate with the development of long-term LEL. Although obesity is a well-documented independent risk factor for lymphedema,^47^ most studies in this review did not report BMI of the patient population. Among the studies that did, Cirik et al.^26^ found greater LEL prevalence among those with BMIs >30 kg/m^2^. The impact of obesity on LEL rates following inguinal lymphadenectomy, however, remains to be thoroughly studied in the literature.

### Describing lymphedema

Although this review was not designed to analyze the diagnostic methods of lymphedema, the heterogeneous ways in which LEL was described across the studies cannot be ignored. The onset of lymphedema following lymphadenectomy is typically characterized by slow progressive swelling of the lower extremity, whereby patients may experience aching pain, heaviness, or tightness.^49^ Chronic lymphedema was assumed by Baur et al.^21^ when the patients showed typical signs during physical examination or presented with complaints, such as swollen legs. Swelling may be referred to as “soft” or “pitting,” in which excess interstitial fluid is displaced in response to applying local pressure.^47^ Minor edema was considered by Bevan-Thomas et al.^25^ as mild to moderate pitting, whereas major edema interfered with the patients’ ambulation.

Circumferential measurements and volume estimations of the lower extremities are among the primary methods used to describe LEL severity. Lymphedema is considered by the American Physical Therapy Association as “mild” if the maximum difference between the affected and contralateral limb is <3 cm, “moderate” if the difference is between 3-5 cm, and “severe” >5 cm.^50^, ^51^ According to the International Society of Lymphology (ISL), LEL is considered mild if the volume differences between limbs are <20%, moderate between 20-40%, and severe if >40%.^51^ LEL was defined by Rageh et al.^36^ as a difference of ≥7% in all circumferential measurements taken of the lower extremity. Although Rodríguez-Trujillo et al.^38^ considered that LEL was present when the limb circumference or volume increased one year after lymphadenectomy, no methods were described on how the measurements were taken. Standardizing definitions and measurement methods for LEL severity could aid physicians’ decisions on how best to manage lymphedema.

### Management of lower extremity lymphedema

Although LEL can be successfully managed with conservative treatment measures, it cannot be cured. Management typically consists of a combination of therapeutic options aimed at improving patient comfort and function by reducing the limb volume.^8^, ^9^, ^10^ Compression therapy with various bandages and compression garments, as well as physical therapy with lymphatic drainage and decongestive therapy are best administered in clinics with therapists trained in lymphedema treatment. Some studies in this review considered lymphedema in patients who were referred to such clinics by their physician.^23^^,^^29^ Other studies reported lymphedema in patients who required elastic bandages, compression stockings, or pneumatic compression therapy.^32^^,^^37^

A growing body of evidence supports the efficacy of microsurgical intervention in the treatment of secondary lymphedema. Despite the need for more long-term research, postoperative outcomes between LVA and VLNT appear to be similarly positive for reduction in limb volume and patient-reported gains in function.^52^, ^53^, ^54^ Preliminary reports of prophylactic microsurgical interventions suggest that LVA could also be an effective strategy aimed at preventing secondary lymphedema following lymphadenectomy.^55^ Further investigation is needed to elucidate in which patients LVA or VLNT could be a feasible prophylactic option with the greatest benefit. No studies in this literature review reported any prophylactic or therapeutic microsurgical interventions for LEL in patients who underwent inguinal lymphadenectomy.

### Strengths and limitations

Although the retrospective nature of several studies analyzed in this review allowed for the inclusion of large sample sizes across several years, it also proved to be a significant limitation. The quality and completeness of data retrieved from the medical records were heterogeneous. Some studies did not report the BMI of the participants^22,^^23^^,^^25^^,^^28^^,^^31^^,^^33^, ^34^, ^35^, ^36^^,^^38^, ^39^, ^40^^,^^42^^,^^43^, which could have further elucidated the impact of obesity on LEL following lymphadenectomy. Furthermore, data retrieved from medical records did not always disclose how lymphedema was defined or measured. Studies that did describe how lymphedema was considered were not consistent in the definitions and measurements employed. This was reflected in the number of studies that either reported LEL prevalence among patients^21^^,^^24^^,^^26^^,^^28^^,^^30^^–^^32^^,^^34^^,^^37^^,^^38^ or per operated groin^22^^,^^23^^,^^25^^,^^27^^,^^29^^,^^33^^,^^35^^,^^36^^,^^39^, ^40^, ^41^, ^42^, ^43^. Although the *p*-value of the *Q-*test between these groups was not statistically significant, results still indicated a difference on a trend level. Similar results were observed for subgroup analysis of studies according to their risk of bias. Studies with high risk of bias^21^^,^^29^^,^^40^ were evaluated as such using the ROBINS-E tool when the complete data on LEL were not available for all participants who underwent inguinal lymphadenectomy. As the missing data could have considerably influenced the difference in the estimated effect of exposure on the outcome, the overall risk of bias in these studies was very high. To produce the most accurate estimation of LEL following inguinal lymphadenectomy, common units of measurement across studies and sufficient follow-up of all study participants are needed.

## Conclusion

The prevalence of LEL in patients who undergo inguinal lymphadenectomy is estimated to be 24%. A higher prevalence was identified in patients for whom inguinal lymphadenectomy was indicated as a treatment for vulvar cancer. Patients over the age of 65 years may also be at a greater risk for developing LEL following inguinal lymphadenectomy. Awareness among physicians of the prevalence of LEL and characteristics of those most at risk could improve patient counseling and strengthen how thoroughly follow-up assessments are conducted. This information can help patient selection for preventive and therapeutic microsurgical interventions by identifying those at the greatest risk for developing LEL.

## Funding

None.

## Ethical approval

Not required.

## Conflict of interest

No authors of this study report disclosure of any commercial or personal interest in the subject of study or of the source of any financial or material support that could bias this work.

## Appendix Supplementary materials

Image, application 1

Image, application 2
