# Supplementary material for: Prevalence of lower extremity edema following inguinal lymphadenectomy: A systematic review and meta-analysis
Source: JPRAS Open. 2024 Nov 17;43:187–99. doi: 10.1016/j.jpra.2024.11.001 (PMC11699470; doi:10.1016/j.jpra.2024.11.001)
Supplement: Supplementary file 2 [file mmc2.docx]

PubMed:

(“lymphedem*”[Title/Abstract] OR “lymphoedem*”[Title/Abstract] OR “edem*”[Title/Abstract] OR “oedem*”[Title/Abstract]) AND ("lymph node dissect*"[Title/Abstract] OR “LND”[Title/Abstract] OR “lymphadenectom*”[Title/Abstract] OR “ILND”[Title/Abstract]) AND (“groin*”[Title/Abstract] OR “inguin*”[Title/Abstract] OR "lower extrem*"[Title/Abstract] OR "lower limb*"[Title/Abstract] OR “leg”[Title/Abstract] OR “legs”[Title/Abstract] OR “thigh*”[Title/Abstract])

OR

("Lymphedema"[Mesh]) AND ("Lymph Node Excision"[Mesh]) AND ("Groin"[Mesh]) AND ("Lower Extremity"[Mesh])

Embase:

(‘lymphedem*’:ti,ab,kw OR ‘lymphoedem*’:ti,ab,kw OR ‘edem*’:ti,ab,kw OR ‘oedem*’:ti,ab,kw) AND ("lymph node dissect*":ti,ab,kw OR ‘LND’:ti,ab,kw OR ‘lymphadenectom*’:ti,ab,kw OR ‘ILND’:ti,ab,kw) AND (‘groin*’:ti,ab,kw OR ‘inguin*’:ti,ab,kw OR "lower extrem*":ti,ab,kw OR "lower limb*":ti,ab,kw OR ‘leg’:ti,ab,kw OR ‘legs’:ti,ab,kw OR ‘thigh*’:ti,ab,kw)

Web of Science:

TI=((“lymphedem*” OR “lymphoedem*” OR “edem*” OR “oedem*”) AND ("lymph node dissect*" OR “LND” OR “lymphadenectom*” OR “ILND”) AND (“groin*” OR “inguin*” OR "lower extrem*" OR "lower limb*" OR “leg” OR “legs” OR “thigh*”))

OR

AB=((“lymphedem*” OR “lymphoedem*” OR “edem*” OR “oedem*”) AND ("lymph node dissect*" OR “LND” OR “lymphadenectom*” OR “ILND”) AND (“groin*” OR “inguin*” OR "lower extrem*" OR "lower limb*" OR “leg” OR “legs” OR “thigh*”))

OR

AK=((“lymphedem*” OR “lymphoedem*” OR “edem*” OR “oedem*”) AND ("lymph node dissect*" OR “LND” OR “lymphadenectom*” OR “ILND”) AND (“groin*” OR “inguin*” OR "lower extrem*" OR "lower limb*" OR “leg” OR “legs” OR “thigh*”))
